# The *Synechocystis* sp. PCC 6803 Genome Encodes Up to Four 2-Phosphoglycolate Phosphatases

**DOI:** 10.3389/fpls.2018.01718

**Published:** 2018-11-27

**Authors:** Snigdha Rai, Stefan Lucius, Ramona Kern, Hermann Bauwe, Aaron Kaplan, Joachim Kopka, Martin Hagemann

**Affiliations:** ^1^Department of Plant Physiology, University of Rostock, Rostock, Germany; ^2^Centre of Advanced Study in Botany, Banaras Hindu University, Varanasi, India; ^3^Department of Plant and Environmental Sciences, The Hebrew University of Jerusalem, Jerusalem, Israel; ^4^Applied Metabolome Analysis, Department of Molecular Physiology, Max Planck Institute of Molecular Plant Physiology, Potsdam, Germany; ^5^Department Life, Light and Matter, University of Rostock, Rostock, Germany

**Keywords:** cyanobacteria, 2-haloacid dehalogenase, mutant, phosphoglycolate phosphatase, photorespiration, *Synechocystis*

## Abstract

Photorespiratory phosphoglycolate (2PG) metabolism is essential for cyanobacteria, algae, and plants. The first enzyme of the pathway, 2PG phosphatase (PGPase), is known from plants and algae but was scarcely investigated in cyanobacteria. *In silico* analysis revealed four candidate genes (*slr0458*, *slr0586*, *sll1349*, and *slr1762*) in the genome of the model cyanobacterium *Synechocystis* sp. PCC 6803 that all belong to the 2-haloacid dehalogenase (HAD) superfamily and could possibly encode PGPase proteins. However, in contrast to known algal and plant PGPases, the putative cyanobacterial PGPases belong to another HAD subfamily implying that PGPases in eukaryotic phototrophs did not originate from cyanobacterial PGPases. To verify their function, these four genes were inactivated both individually and in combination. A mild high-CO_2_-requiring (HCR) growth phenotype typical for photorespiratory mutants was observed only in Δ*sll1349*. Combinatorial inactivation enhanced the HCR phenotype in specific double and triple mutants. Heterologous expression of the putative cyanobacterial PGPases in *E. coli* led to higher PGPase activities in crude cell extracts, but only the purified Slr0458 protein showed PGPase activity. Hence, we propose that a consortium of up to four photorespiratory PGPases may initiate photorespiratory 2PG metabolism in *Synechocystis*. We suggest that redundancy of this essential enzyme activity could be related to the highly adaptive lifestyle of cyanobacteria such as *Synechocystis* sp. PCC 6803, which allows them to grow under very diverse conditions.

## Introduction

All oxygenic phototrophs use the enzyme ribulose-1,5-bisphosphate carboxylase/oxygenase (Rubisco) for carbon assimilation. However, Rubisco catalyzes two competing reactions, carboxylation and oxygenation of ribulose-1,5-bisphosphate (RuBP), the ratio of which mainly depends on the concentrations of CO_2_ and O_2_. Oxygenation of RuBP produces 2PG, a compound that inhibits Calvin-Benson cycle enzymes (e.g., triosephosphate isomerase, Husic et al., 1987; sedoheptulose 1,7-bisphosphate phosphatase, Flügel et al., 2017), lowering the rate of photosynthesis. Therefore, 2PG is rapidly metabolized by the photorespiratory pathway (Tolbert, 1997; Bauwe et al., 2010), which recovers one molecule of 3-phosphoglycerate from two molecules of 2PG while one carbon atom is lost as photorespiratory CO_2_. Moreover, the conversion of two molecules of glycine into serine releases NH_3_. In addition to these functions, detoxification and organic carbon salvage, it has been suggested that photorespiration provides glycine and serine to cellular metabolism and helps protecting from photoinhibition and oxidative damage (e.g., Wingler et al., 2000).

It has been established that there is no oxygenic photosynthesis without photorespiration (Bauwe et al., 2012). Accordingly, the inactivation of photorespiration-related genes in C_3_ plants typically produces mutants that show a HCR phenotype (Somerville, 2001; Timm et al., 2012). To deal with limiting CO_2_ amounts, cyanobacteria, many algae, and C_4_ plants evolved inorganic carbon concentrating mechanisms (CCM; Giordano et al., 2005), which were believed to make photorespiration superfluous. However, *Chlamydomonas* and maize mutants with intact CCM but defective photorespiration also showed the HCR photorespiratory phenotype (Nakamura et al., 2005; Zelitch et al., 2009). Photorespiration is also essential for cyanobacteria despite their CCM (Eisenhut et al., 2008b). All studied cyanobacteria, even those with small genome size as the picoplanktonic *Prochlorococcus* and *Synechococcus* spp., have preserved complete and active sets of photorespiratory genes (Kern et al., 2011). Among cyanobacteria, most of the functional analysis of photorespiration has been performed in the model strain *Synechocystis* sp. PCC 6803 (hereafter *Synechocystis*), which possesses three different routes for 2PG metabolism: plant-like C2 pathway, bacterial glycerate pathway, and complete decarboxylation of glyoxylate (Eisenhut et al., 2008b). The photorespiratory intermediate glycolate is produced in *Synechocystis* WT cells even at 5% CO_2_ (Huege et al., 2011) suggesting that operation of the CCM is not sufficient to completely suppress RuBP oxygenation. Due to the similarity of photorespiratory enzymes in plants and cyanobacteria and the essential relation between photorespiration and photosynthesis, we hypothesized that the photorespiratory pathway might have evolved very early among cyanobacteria, before the primary endosymbiosis leading to the first photosynthetic eukaryotes (Eisenhut et al., 2008b).

Many enzymes of the cyanobacterial photorespiratory metabolism have been studied during the last few years (e.g., Eisenhut et al., 2008b; Hackenberg et al., 2011), but PGPase (EC 3.1.3.18) and the corresponding gene(s) were only scarcely investigated (Haimovich-Dayan et al., 2015). PGPase catalyses the entry reaction into photorespiratory 2PG metabolism: dephosphorylation of 2PG to glycolate. The enzyme belongs to the HAD protein superfamily that possesses three conserved motifs: motif I, DX(D/T/Y)X(T/V)(L/V); motif II, (S/T); and motif III, K(G/S)(D/S)XXX(D/N). An aspartate (D) at position three in motif I is characteristic for phosphatases. During catalysis, motif I is self-phosphorylated, motif II forms hydrogen bonds with the substrate, and motif III interacts with divalent metal ions at the active site (Kim et al., 2004). PGPases have been purified from plants such as tobacco, spinach, maize, pea; and the green alga *Chlamydomonas* as well as the cyanobacterium *Coccochloris* (Kerr and Gear, 1974; Husic and Tolbert, 1984; Hardy and Baldy, 1986; Norman and Colman, 1991). Pellicer et al. (2003) compared the structural and functional features of PGPases of different origins and found similarity of the sequences, implying the use of the same catalytic mechanism. However, the bacterial proteins belong to the dehr (dehalogenase-related)-like protein family, whereas PGPases from eukaryotic phototrophs are members of the NagD (ribonucleotide monophosphatase)-like protein family among the HAD protein superfamily (Burroughs et al., 2006). PGPase mutants have been isolated from Arabidopsis (Somerville and Ogren, 1979; Schwarte and Bauwe, 2007) and *Chlamydomonas* (Suzuki et al., 1999; Ma et al., 2013). All of them were reported to exhibit the characteristic HCR phenotype (Timm et al., 2012; Flügel et al., 2017).

Here, we report on the identification of *Synechocystis* PGPases. By using the basic local alignment search tool (BLAST), we found four candidate genes (*slr0458*, *slr0586*, *sll1349*, and *slr1762*). The four candidate cyanobacterial genes encode for proteins of the HAD superfamily (Supplementary Figure S1) but contrary to known algal and plant PGPases, the putative cyanobacterial PGPases belong to another HAD subfamily implying that PGPases in eukaryotic phototrophs did not originate from the cyanobacterial proteins. Their function was studied *in vitro* after expression in *E. coli* and by the analysis of single and combined *Synechocystis* mutants. We conclude that a consortium of up to four photorespiratory PGPases may initiate photorespiratory 2PG metabolism in *Synechocystis*.

## Materials and Methods

### Strains and Culture Conditions

The glucose-tolerant *Synechocystis* sp. strain PCC 6803 served as WT. Axenic cultures were grown photo-autotrophically in batch cultures (3 cm glass vessels with 5 mm glass tubes) by bubbling with air (flow rate 5 mL min^-1^) enriched with CO_2_ (5% CO_2_, defined as HC) at 29°C under continuous illumination of 100 μmol photons m^-2^ s^-1^ (warm white light; Osram L58 W32/3) in BG11 medium (Rippka et al., 1979) buffered with TES-KOH (20 mM final concentration) at pH 8.0. Cultures at an optical density at 730 nm (OD_730_) of approximately 1.0 (equivalent to approximately 10^9^ cells mL^-1^) was used for the experiments. Shifts to ambient air conditions (low CO_2_ of 0.04%, LC) were performed by switching the bubbling from CO_2_-supplemented to air as described previously (Hackenberg et al., 2009).

### Construction of Single and Multiple Mutants

To generate single and multiple mutations in the selected genes, interposon mutagenesis was applied by insertion of antibiotic resistance cassettes into the coding sequences at selected restriction sites (e.g., Bryant et al., 1990). The primers were designed for the coding sequences and neighboring sequences (approximately 2 kb length) using the complete genome sequence of *Synechocystis* (Kaneko et al., 1996). Supplementary Table S1 includes the respective list of antibiotic cassettes used for each gene and the primers. Total DNA from *Synechocystis* was isolated according to Hagemann et al. (1997).

The inactivated genes for *slr0458* and *sll1349* were amplified via PCR from the previously used mutants of *Synechocystis* (Eisenhut et al., 2006) and ligated into pGEMT (Promega). To inactivate *slr0586*, the coding sequence and flanking DNA was obtained from the *Synechocystis* WT and ligated into pGEMT. The coding sequence was cut with *Mun*I and a blunt-ended erythromycin resistance cartridge was inserted. A clone harboring the resistance gene in the same transcriptional orientation as *slr0586* was used for the transformation of the *Synechocystis* WT. The coding sequence of gene *slr1762* together with 800 bp flanking sequence was amplified from WT DNA and ligated into pGEMT. The central part of the coding sequence was deleted by restriction with *Eco*O109I and after generating blunt ends it was replaced by the spectinomycin resistance cassette.

All mutation constructs were verified by restriction analysis and sequencing (Seqlab, Germany). Positive constructs were used for transforming *Synechocystis* as described previously (Hagemann and Zuther, 1992). Transformation, selection, and segregation of mutants were done in the presence of 5% CO_2_ to decrease selective pressure from photorespiratory activity. Mutant colonies were screened against the corresponding antibiotics added to the petri dishes containing BG11 with 0.8% Kobe agar. Drug-resistant colonies were transferred on fresh plates to allow complete segregation of the *Synechocystis* genome. The constructs for generating single mutations in *slr0458*, *slr0586*, and *sll1349* were subsequently used to transform the *Synechocystis* mutant*Δslr1762* for the second and third transformations raising double and triple gene mutants, respectively.

All mutants were further stored and cultivated under HC conditions. To show alterations in the genotype, PCR with gene-specific oligonucleotides (Supplementary Table S1) was carried out by using the Taq-PCR Master Mix (Qiagen) as described in Eisenhut et al. (2006). Mutant colonies showing complete segregation of the target gene, only DNA fragment with sizes corresponding to the mutated genes in the absence of the respective WT gene bands (see Supplementary Figure S2), were used for further experiments.

### Phenotypic Characterization of *Synechocystis* Mutants

For physiological and molecular phenotyping, exponentially growing cultures (OD_730_ = 0.5) were pre-cultured at HC conditions as described by Hackenberg et al. (2009). These cells were then further grown in the Multi-Cultivator MC 1000 (Photon System Instruments, Czech Republic) by bubbling with ambient air (LC) at 150 μmol photons m^-2^ s^-1^ light and 30°C. The MC1000 allows an automated measurement of growth as optical density of cultures at 730 nm (OD_730_). The optical density was recorded every 30 min during a growth period of 5 days. Periods of exponential growth were used to calculate specific growth rates. In each experiment, the WT and selected mutants were grown in duplicates to compare the specific growth rates. The experiments were repeated at least 2-times. Statistically significant differences in the growth rates between the WT and the mutant strains were analyzed using 1-way ANOVA. Cultures, pre-grown in 5% CO_2_ with OD_730_ = 0.5 were also used for drop dilution assay tests to search for the HCR phenotype. WT and mutant cultures were serially diluted 10, 100, 1000, and 10,000 fold; 2 μl of these dilutions were spotted on BG11-Kobe agar plates, allowed to dry and incubated in ambient air at similar temperature and light conditions as used for the cultivation of liquid cultures.

### Metabolite Profiling for Selected Mutants

Cells grown in BG11 liquid media under LC culture conditions were harvested by fast filtration and immediately frozen in liquid nitrogen as described by Eisenhut et al. (2008a). The mutants were grown in duplicates and at least three replicates from each culture were analyzed in all cases. Steady state contents of metabolites, e.g., 2PG and glycolate, in cyanobacterial cells were determined using non-targeted metabolite profiling by gas chromatography-electron impact-time of flight-mass spectrometry (GC-EI-TOF-MS). Amounts of metabolites were normalized to biomass using the OD_730_ of each sample (Eisenhut et al., 2008a; Huege et al., 2011) and to the internal standard U^13^C-sorbitol using the normalization procedure described by Lisec et al. (2011). Analytes were identified by at least 3 specific mass fragments per compound and a retention index deviation < 1.0% (Hummel et al., 2010). Metabolites were assessed by relative changes expressed as response ratios, i.e., x-fold factors of mutant cells in comparison to the WT that was arbitrarily set to 1. Statistical analysis was performed using the heteroscedastic Student’s *t*-test in Excel 2010. The complete data set is shown in Supplementary Table S3.

### Cloning of Genes for Overexpression in *E. coli*

For overexpression of the candidate genes, the pET28a Expression System (Novagen) was used. Open reading frames of all the four genes (*slr0458*, *slr0586*, *sll1349*, and *slr1762*) were amplified from *Synechocystis* WT chromosomal DNA by PCR using Taq polymerase master mix (Qiagen) and elongase enzyme mix (Thermo Fisher Scientific). Gene-specific primers (Supplementary Table S1) contained added *Nde*I and *Eco*RI restriction sites at the 5′ and 3′ ends, respectively, to facilitate cloning of cyanobacterial genes in frame with the N-terminal His-tag. Before the ligation with pET28a, the PCR fragments were cloned into pGEMT (Promega) and the inserts were verified by sequencing. Correct inserts were then excised with *Nde*I and *Eco*RI, ligated into pET28a and transferred into *E. coli* strain BL21 DE3 (Novagen). The cells were cultured at 37°C at 170 rpm shaking in LB medium until the suspension reached an OD_600_ of 0.5. Cultures with empty vector were simultaneously maintained as control. Gene expression was induced by addition of 0.5 mM IPTG and the cells were then cultured overnight at room temperature. Soluble proteins were extracted from *E. coli* by sonication with homogenization buffer (20 mM Tris buffer, 500 mM NaCl, pH 7.8) and centrifugation (40,000 ×*g*, 4°C). His-tagged recombinant proteins were purified by affinity chromatography on Ni-NTA columns (ProBond, Thermo Fisher Scientific) and eluted with gradually increasing imidazole concentrations. The purity of recombinant proteins was confirmed by SDS-PAGE (see Supplementary Figure S4). For comparison, the recombinant PGLP1 (At5g36700) from Arabidopsis was obtained as described by Schwarte and Bauwe (2007).

### Protein Quantification and PGPase Activity

His-tag-purified recombinant proteins and soluble protein extracts from *E. coli* were used for the PGPase assay. Total protein concentrations of all samples were determined according to Bradford (1976) and enzymatic assays (*N* = 3) performed as described in Schwarte and Bauwe (2007). In short, assays were incubated for 10 min at 37°C with 2 mM 2PG (Sigma-Aldrich). Aliquots taken every 2 min were assayed for inorganic phosphate via the formation of reduced phosphomolybdate (Ames, 1966). A set of different Na_2_HPO_4_ concentrations was used for calibration.

### Phylogenetic Analyses

The sequences used for the alignment were identified via BlastP (Altschul et al., 1990) using the Arabidopsis PGPase (At5g36700; Schwarte and Bauwe, 2007) and the *Synechocystis* PGPase candidate proteins Slr0586, Slr1762, Sll1349 or Slr0458 as template. The BlastP searches were limited to specific taxonomic groups (e.g., cyanobacteria). In addition to plant and cyanobacterial proteins, we also included verified PGPases from the bacteria *Ralstonia eutropha*, *Escherichia coli* and *Rhodobacter sphaeroides* (Schäferjohann et al., 1993; Gibson and Tabita, 1997; Lyngstadaas et al., 1999). The sequences were aligned using the high accuracy algorithm Probcons (Do et al., 2005) implemented at the MPI TOOLKIT platform (Biegert et al., 2006). Thereafter, putative chloroplast target peptides and other miss-aligned regions in the C- or N-terminus were removed. According to the Akaike information criterion calculated via ProtTest 3.2 (Darriba et al., 2011), the best fitting model is the LG+G+I (amino acid replacement matrix from Le and Gascuel, 2008; including a proportion of invariant sites and a gamma model of rate heterogeneity). After visual inspection, the alignment was used for phylogenetic reconstruction using Bayesian interference implemented in MrBayes 3.2 (Ronquist et al., 2012) running 2,000,000 generations, whereas the first 25% of samples were disregarded as burn-in. Additionally, Maximum Likelihood with 1000 bootstrap replicates implemented in RAxML (Stamatakis, 2014) was applied. The phylogenetic tree was visualized using FigTree v1.4.2^1^. The alignments can be found as Supplementary Figure S5. Species names and accession numbers are as follows: *Microcystis aeruginosa* PCC 9701 (CCI35500.1, WP_002803420.1, CCI39026.1), *Microcoleus vaginatus* FGP-2 (WP_006635473.1, EGK86926.1, EGK83991.1), *Synechocystis* sp. PCC 6803 (Slr0586, Slr1349, Slr1762, Slr0458; CyanoBase), *Nostoc* sp. PCC 7120 (Alr3258, Alr4944, All0135; CyanoBase), *Synechocystis* sp. PCC 6714 (WP_028946651.1), *E. coli* O157:H7 (P58422.1), *R. eutropha* (AAA20197), *R. sphaeroides* ATCC 17025 (A4WW31.1), *Arabidopsis thaliana* (At5g36700; TAIR), *Physcomitrella patens* (XP_001758778), *Zea mays* (ACF84012). If not otherwise mentioned sequences were retrieved from NCBI.

## Results

### Phylogenetic Analysis of PGPase Proteins

BlastP searches with the Arabidopsis PGPase (PGLP1, At5g36700) in the *Synechocystis* genome identified Slr0458, Slr0586, Sll1349, and Slr1762 as candidates for putative cyanobacterial PGPases. These proteins possess HAD family domains, a characteristic feature of functional PGPases (Supplementary Figure S1). Notably, the four proteins showed only a low degree of similarity (48–65%) to each other and with At5g36700 (23–32%; Supplementary Table S2). Among the *Synechocystis* PGPase candidate genes, only *sll1349* is preliminarily annotated as PGPase in CyanoBase^2^, whereas the other three proteins are annotated as hypothetical or unknown proteins. Within the HAD superfamily, all cyanobacterial proteins belong to the dehr (dehalogenase-related)-like protein family, whereas PGLP1 of Arabidopsis is a member of the NagD (ribonucleotide monophosphatase)-like protein family (Burroughs et al., 2006).

**FIGURE 1:**
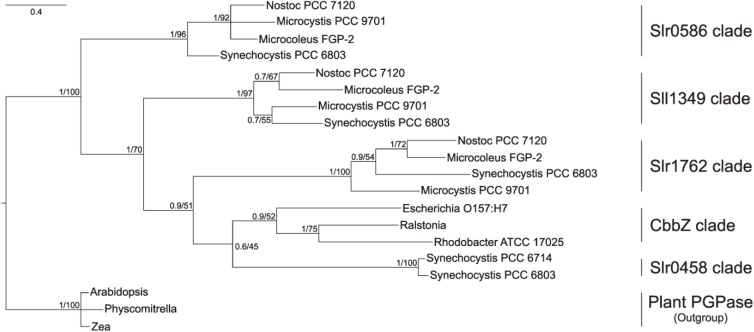
Phylogenetic tree of cyanobacterial PGPase candidate proteins showing relationship of *Synechocystis* PGPases with proteins from other cyanobacteria, bacteria, and plants. The tree was constructed using Bayesian interference running 2,000,000 generations whereas the first 25% were sort as burn-in. Numbers at the node indicate Bayesian post probabilities and Maximum likelihood bootstrap support values, respectively. Species names and accession number are given in the Material and methods section.

In the phylogenetic tree (Figure 1), all bacterial proteins belonging to the dehr-like protein family grouped into on large clade, which is clearly separated from the plant proteins of the NagD-like family. The four candidate proteins from *Synechocystis* are separated into four different subclades. The Slr0458 protein forms one clade together with the bacterial CbbZ clade, which comprises well characterized PGPases from bacteria such as *E. coli*, *R. sphaeroides*, and *R. eutropha* H16. The Slr1762–like cyanobacterial homologs are situated next to this clade. The Sll1349 homolog in *Synechococcus* PCC 7942 is also annotated as *cbbZ*^3^, however, Sll1349-like proteins cluster separately from the CbbZ-Slr048-Slr1762 clades. The Slr0586 protein and homologs from other cyanobacterial strains build a third clade (Figure 1). The plant PGPase proteins, e.g., PGLP1 (At5g36700) of Arabidopsis, form a separate group together with PGPases from algae and were used as outgroup (Figure 1).

BLAST searches against complete cyanobacterial genomes listed in CyanoBase^4^ revealed that Slr0586, Slr1762, and Sll1349 proteins have homologs in many other cyanobacteria (80–60 other strains), which all are annotated as HAD-like proteins. Remarkably, the closest CbbZ-homolog Slr0458 is only found in the genome of *Synechocystis* and its closely related strain *Synechocystis* sp. PCC 6714 (Kopf et al., 2014). It needs to be mentioned that we did not find similar proteins in any of the genomes from strains of the marine *Synechococcus*/*Prochlorococcus* clades.

Thus, the cyanobacterial PGPase candidates are not homologous to the well characterized enzyme from Arabidopsis (Schwarte and Bauwe, 2007). To verify whether these proteins are PGPases, we generated insertion mutants affected in single and multiple genes, which were characterized regarding their ability to grow and accumulate 2PG under LC conditions. The candidate genes were also cloned into *E. coli* expression vectors to obtain recombinant proteins to verify the PGPase activity *in vitro*.

### Generation of Single, Double, and Triple *Synechocystis* Mutations

**FIGURE 2:**
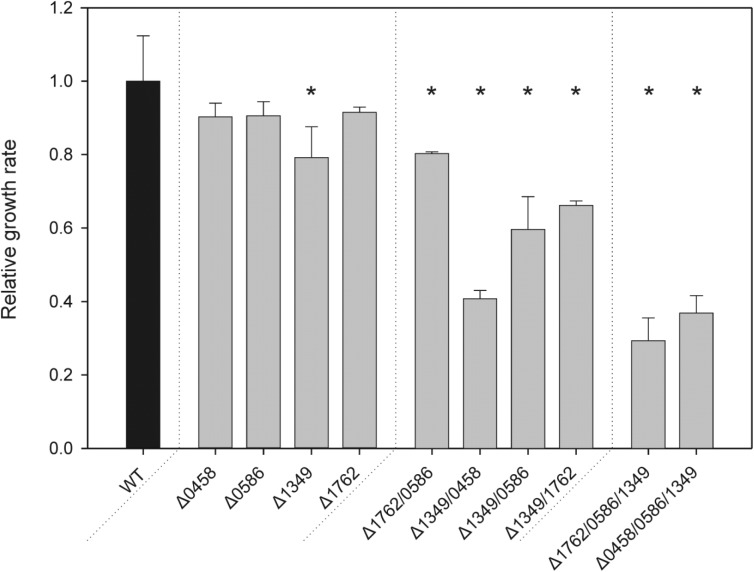
Specific growth rates of PGPase mutants as compared to the wild type (WT). Different sets of single, double, or triple mutants (gray bars) were grown side by side with the WT(black bar) under LC conditions. Given values are means ± SD (*n* = 3) and asterisks indicate values statistically different from the control (^∗^*p* < 0.05, 1-way ANOVA). The growth of the WT was set to 1 and corresponds to a growth rate of 0.017 h^-1^.

To analyze the function of the four candidate proteins, we first inactivated each coding sequence via insertion of different drug resistance cartridges, which allowed the subsequent combination of the gene defects. PCR analyses showed that all WT copies of the respective genes were inactivated (Supplementary Figure S2). The complete segregation of the mutants revealed that under HC (5% CO_2_ in air) conditions none of the genes is essential for viability particularly since glycolate, the immediate product of PGPase, was also detected under HC (Huege et al., 2011). The mutants were then subjected to LC (0.04% CO_2_) conditions, where all of the single mutants were able to grow. The growth was comparable to WT in drop dilution assays on solid media (Supplementary Figure S3). Comparing specific growth rates in liquid media (Figure 2), three single mutants showed no significant changes compared to WT at LC, whereas mutant *Δsll1349* showed slower growth, i.e., has a weak HCR phenotype. This finding indicates that more than one functional PGPase exists in *Synechocystis*. Hence, we generated a set of various double mutants. Again, it was possible to obtain completely segregated clones of *Synechocystis* mutants in which different pairs of putative PGPase-encoding genes were disrupted under HC conditions. All generated double mutants were able to grow at LC on plates (Supplementary Figure S3). However, the estimated growth rates in liquid cultures revealed differences in the performances under LC conditions. Compared with the WT, the double mutant *Δsll1349*/*slr0458* showed a 60%, *Δsll1349*/*slr0586* a 41%, *Δsll1349*/*slr1762* a 34%, and *Δslr0586*/*slr1762* a 17% decrease in specific growth rates in LC (Figure 2). An even stronger HCR phenotype was observed with the triple mutants *Δslr1762/slr0586/sll1349* and *Δsll1349/slr0586/slr0458*. These mutants also seem to grow ∼50% slower than WT at HC conditions, because it took more than 3 weeks before clones of triple mutants appeared on agar plates, whereas other clones already appeared after 1 week. At LC conditions, a clear decline of growth was observed for the triple mutants. The growth rate of *Δslr1762/slr0586/sll1349* was dropped by approximately 70% of WT when cultivated at LC. A similar growth decline was observed for another triple mutant, *Δsll1349/slr0586/slr0458.* Notably, several attempts to generate the respective quadruple mutant failed, we could not obtain mutant colonies even under HC conditions.

### Accumulation of 2PG in Mutant Cells

**FIGURE 3:**
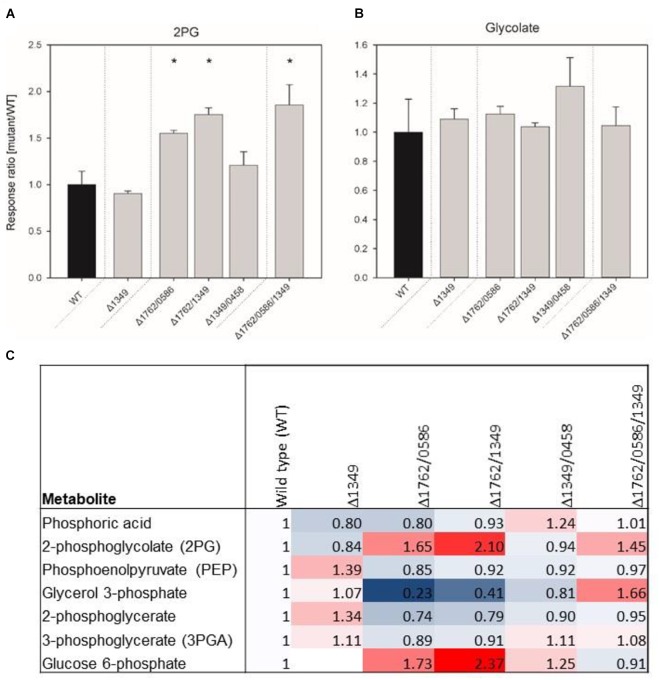
Relative contents of 2-phosphoglycolate (2PG, **A**), glycolate **(B)** and several other phosphorylated compounds **(C)** in mutant and wild type (WT) cells of *Synechocystis*. The data represent response ratios and correspond to averaged x-fold values of three biological replicates measured by at least two technical replicates. Factors are calculated relative to WT. Error bars represent standard error. Significant differences to the WT are marked by asterisks (^∗^*p* < 0.05; Student’s *t*-test).

The growth experiments showed gradually decreasing growth rates, starting from single over double to triple mutants, which could be associated with increased 2PG levels if the enzymes have PGPase activity. Thus, mutant gene combinations Δ*sll1349*, Δ*slr1762*/*slr0586*, Δ*sll1349*/*slr176*2, Δ*sll1349*/*slr0458*, Δ*sll1349*/*slr0586*, and Δ*sll1349*/*slr0586*/*slr1762* were selected for profiling of the intracellular concentrations of 2PG and glycolate after cultivation at LC conditions (Figure 3). No major changes in 2PG levels were observed in the cells of the tested single mutant Δ*sll1349*. Cells of the double mutant Δ*slr1762*/*slr0586* and Δ*sll1349*/*slr176*2 showed 55 and 77% increased accumulation of 2PG compared to WT cells, whereas the double mutant Δ*sll1349/0458* only slightly increased the internal 2PG amounts (Figure 3A). Highest accumulation of 2PG, 86% increase compared to the WT was recorded for the triple mutant Δ*slr1762*/*slr0586*/*sll1349* (Figure 3A) In contrast to 2PG, only small amounts of glycolate were found in all cells, which are largely unaltered in all mutants compared to WT (Figure 3B). Generally, the 2PG accumulation is consistent with the previous findings of declined growth of double and triple mutants at LC conditions.

Some additional changes were found in the metabolome data set regarding phosphorylated intermediates (Figure 3C). In addition to 2PG other phosphorylated compounds showed changed levels in mutant cells. For example, up to twofold higher amounts of glucose 6-phosphate were found in cells of double mutants, whereas the 3PGA level decreased compared to WT cells in these mutants.

### PGPase Activities of Recombinant Proteins

**FIGURE 4:**
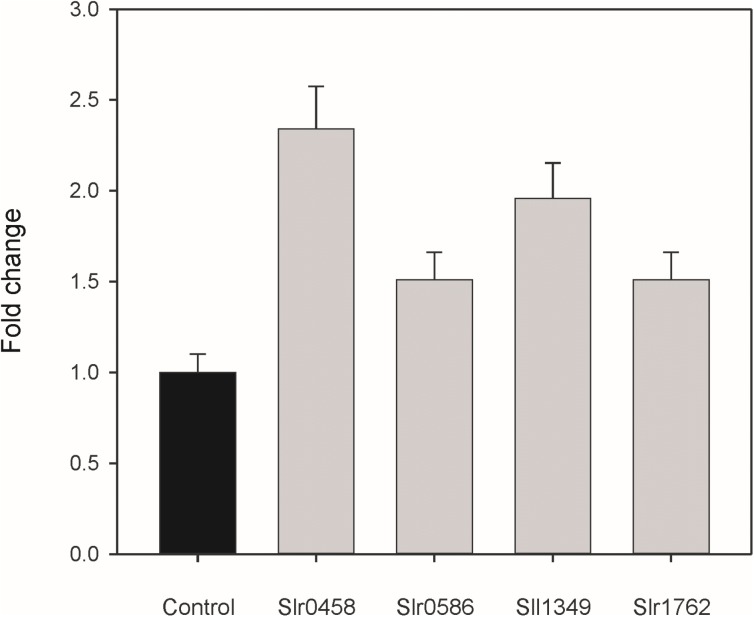
PGPase activities of recombinant proteins using 2PG as substrate. The putative PGPase from *Synechocystis* were expressed as tagged proteins in *E. coli* BL21 cells. Soluble cell extracts were used for enzyme assays in comparison to cells with the empty expression vector (control). The empty vector control showed PGPase activities of 3.1 nmol P_i_ min^-1^ mg^-1^ protein, which were set to 1 and relative values given are means ± SD.

The metabolite profiling data clearly indicated that inactivation of the selected genes results in intracellular accumulation of 2PG. To have direct evidence that the selected *Synechocystis* genes code for active PGPases, they were expressed in *E. coli*. First we analyzed the PGPase activities in total cell extracts of recombinant *E. coli* (Figure 4). Extracts from overexpression strains harboring the cyanobacterial PGPases in the pET28a expression systems showed up to 2 times higher PGPase activity than the extract from the empty vector control (Figure 4). The highest PGPase activity was observed in cells expressing *slr0458* followed by cells expressing *sll1349*. These initial experiments verified that all four candidate proteins from *Synechocystis* are capable of 2PG dephosphorylation.

Next, we went on to isolate purified recombinant proteins. Unfortunately, only three out of the four candidate proteins could be obtained as soluble proteins. Despite many attempts, protein Slr0586 was always found only in the insoluble fraction and therefore could not be tested *in vitro*. The Slr0458 and Sll1349 proteins were obtained in large amounts; whereas the yield of the Slr1762 protein was significantly lower (Supplementary Figure S4). However, all the purified recombinant proteins showed some instability, which became obvious since they started to precipitated when stored on ice for 1 h. The enzyme assays with 2PG (2 mM final concentration) as substrate revealed low specific PGPase activities for Slr0458 (0.54 ± 0.12 μmol min^-1^ mg^-1^) and for Sll1349 (0.23 ± 0.09 μmol min^-1^ mg^-1^). Enzymatic active Slr0458 expressed in *E. coli* was also obtained before by Haimovich-Dayan et al. (2015). The purified recombinant protein Slr1762 showed no PGPase activity at all. For comparison, we included the purified recombinant PGPase from Arabidopsis (PGLP1, At5g36700), which showed high specific PGPase activities of 25.48 ± 1.15 μmol min^-1^ mg^-1^. Thus, the purified plant enzyme has a more than 50 fold higher activity compared to the most active cyanobacterial PGPase Slr0458 in these experiments.

## Discussion

*Chlamydomonas* and land pants such as Arabidopsis harbor single genes encoding for chloroplastidial PGPase functioning in the photorespiratory pathway. Additional cytosolic isoforms are not involved in photorespiration (Schwarte and Bauwe, 2007) but serve to destroy toxic side products from mainline carbon metabolism (Collard et al., 2016), which corresponds with our observation that more than one PGPase are present in the non-compartmented cyanobacterial cell of *Synechocystis*. This conclusion is mainly based on the mutation approach, where two and more genes for the putative PGPases needed to be inactivated before a growth decline and marked 2PG accumulation under LC conditions appeared (see Figures 2, 3). This is in contrast to the finding that inactivation of the single genes encoding for chloroplastidial PGPase of *Chlamydomonas* or Arabidopsis resulted in HCR phenotypes (Suzuki et al., 1999; Schwarte and Bauwe, 2007).

Only the *Synechocystis* single mutant Δ*sll1349* showed a decline in generation time under LC conditions compared to WT. This finding suggests that Sll1349 might represent the most active PGPase in *Synechocystis*. However, the 2PG level was not significantly changed in this mutant strain, therefore, the involvement of the Sll1349 in another important enzymatic reaction can not be ruled out. Among the double mutants, the combination of *sll1349* and *slr0458* disruption resulted in the most severe ( > 50%) growth decline pointing at Slr0458 as the second most active PGPase in *Synechocystis* under the tested growth condition. The protein Sll1349 is already annotated as PGPase in CyanoBase^5^ due to its similarity to CbbZ in different bacteria. However, our phylogenetic analysis (see Figure 1) revealed that the Sll1349 protein clade is rather distantly related to the CbbZ clade. In contrast, clades of Slr0458 and Slr1762-like proteins from cyanobacteria are situated next to the CbbZ clade. The CbbZ group comprises well-characterized PGPases from *R. eutropha, E. coli* and *R. sphaeroides* (Schäferjohann et al., 1993; Gibson and Tabita, 1997; Lyngstadaas et al., 1999). In *Rhodobacter*, the *cbbZ* gene is part of the *cbbI*-operon downstream of the gene for form I Rubisco. Consistent with their functional dependence, the Rubisco and PGPase proteins are co-expressed under photoautotrophic growth conditions (Gibson and Tabita, 1997). The phylogenetically related PGPase of *E. coli* encoded by *gph* also clusters in the CbbZ group (Figure 1). However, this likely PGPase is part of the *dam* containing operon linking it to DNA repair processes in *E. coli* (Lyngstadaas et al., 1999; Pellicer et al., 2003).

Slr1762, Slr1349, and Slr0586 are the *Synechocystis* PGPase having homologous proteins in many other cyanobacteria with known genome sequences, where they probably also function as photorespiratory PGPase. Interestingly, the gene for the Slr1762 homolog in *Synechococcus* *elongatus* PCC 7942 was found among the LC-induced genes (Schwarz et al., 2011). Photorespiratory genes including the PGPases are also up-regulated by LC in *Chlamydomonas* (Fang et al., 2012). However, all four PGPase encoding genes of *Synechocystis* showed similar transcript abundances under different CO_2_ conditions (Eisenhut et al., 2007). Remarkably, Slr0458, which seems to play an important role in 2PG dephosphorylation in *Synechocystis* together with Sll1349, is only found in the closely related cyanobacterium *Synechocystis* sp. PCC 6714 (Kopf et al., 2014).

The absence of a clear photorespiratory phenotype in the *Synechocystis* double and triple mutants is likely due to the activity of the CCM (Burnap et al., 2015), which minimizes RuBP oxygenation and hence 2PG accumulation. Notably, we were unable to obtain a quadruple mutant, in which all four genes were inactivated even under continuous HC conditions. Probably, the low rate of RuBP oxygenation in cells grown at HC (Huege et al., 2011) already results in 2PG levels that are not tolerated by cells lacking PGPase activity. A very severe HCR phenotype was also observed for the Arabidopsis *pglp1* mutant, which does not fully recover at 1% CO_2_, which is in contrast to other plant mutants affected in photorespiratory enzymes (Timm et al., 2012). The growth decline in the different mutant combinations generally correlated with metabolite profiling data, where a considerable rise in 2PG accumulation was observed from single to double mutants with a maximum in the triple mutant (see Figure 3). These results support the notion that the inactivated genes indeed encode for functional PGPases *in vivo* that collectively dephosphorylate 2PG. It has been shown that increased 2PG levels due to lack of efficient 2PG recycling reduce growth of plants, because 2PG inhibits triosephosphate isomerase, phosphofructokinase and sedoheptulose 1,7-bisphosphate phosphatase (Anderson, 1971; Kelly and Latzko, 1976; Flügel et al., 2017). However, the slower growth of the single mutant Δ*sll1349* did not correlate with increased 2PG accumulation. This observation and the alterations in other phosphorylated compounds (see Figure 3C) might indicate that some of these enzymes have broader substrate specificity *in vivo.* Alternatively, the change in other phosphorylated compounds could also be taken as an indication that 2PG might act as a metabolic regulator.

In contrast to the enhanced values of 2PG in PGPase double and triple mutants, all *Synechocystis* mutants including the triple mutant contained almost the same amount of glycolate as WT cells (see Figure 3). However, bearing in mind that a significant glycolate accumulation was only detected in LC-grown cells of *Synechocystis* when the two glycolate dehydrogenases were inactivated (Eisenhut et al., 2008b; Huege et al., 2011), it is likely that the rapid glycolate conversion prevents marked changes in the glycolate pool in the different mutant strains. Hence, we assume that the observed glycolate pool is probably below the affinity of the downstream enzymes, explaining this low and almost constant pool. Nevertheless, the detection of glycolate in all strains supports the notion that more than one PGPase is actively converting 2PG into glycolate in *Synechocystis*.

To directly verify the enzymatic activity of the candidate proteins, we expressed these genes in *E. coli* as tagged proteins for biochemical analysis. Enhanced PGPase activity, compared to cells with empty vector, was observed in extracts from cells expressing these cyanobacterial genes (see Figure 4); however, the purified recombinant proteins Slr0458 and Sll1349 showed only low PGPases activity compared to recombinant PGPase of Arabidopsis. The low specific activity is most probably due to the instability of the cyanobacterial proteins, which could have caused an underestimation of the specific activities of cyanobacterial PGPases in this study. A substantial amount of eluted protein precipitated within 1 h of storage on ice indicating incorrect folding of the recombinant proteins. Similar stability problems were reported in previous attempts to purify the PGPase from cyanobacterial cells (Norman and Colman, 1991) or to obtain Slr0458 as recombinant protein (Haimovich-Dayan et al., 2015). The latter study also showed PGPase activity of Slr0458 in crude extracts of *E. coli*, which was about 4 times higher than the vector control. The instability of enzymes made it impossible to verify the substrate specificity of the four *Synechocystis* PGPase proteins. Since the levels of some other phosphorylated compounds are also changed in cells of different double mutants (see Figure 3C), it is likely that some of these enzymes act on a number of substrates *in vivo*.

## Conclusion

We demonstrate that *Synechocystis* PGPases are not homologous to PGPases in eukaryotic phototrophs. The cyanobacterial proteins belong to the previously defined dehr family of HAD family proteins, whereas the plant enzymes are found in the NagD family (Burroughs et al., 2006). In contrast to all other photorespiratory enzymes, the plant PGPase most probably originated from the archaeal host cell (discussed in more detail in Hagemann et al., 2016) during eukaryogenesis (Spang et al., 2015). Only three cyanobacterial strains of the genera *Leptolyngbya* and *Coleofasciculus* possess genes for PGPase-like proteins of the NagD family that are closely related to the plant PGPases. However, these genes were most probably obtained via inter-bacterial lateral gene transfer, as the cyanobacterial proteins are situated in-between the bacterial clade (see Hagemann et al., 2016). It is interesting to note that the red alga *Galdieria sulphuraria* also harbors a Slr0458-like protein (Accession Number: XP_005708578.1) with more than 40% identical amino acids. It should be mentioned that plant-like PGLP1 proteins are also encoded in red algal genomes. The finding of two putative PGPases in the *G. sulphuraria* genome might indicate that, like cyanobacteria, red algae may use several photorespiratory PGPases, whereas green algae and plants such as *Chlamydomonas* and Arabidopsis (Suzuki et al., 1999; Schwarte and Bauwe, 2007) use only one photorespiratory PGPases in the chloroplast. The use of multiple PGPases among cyanobacteria could be related to the highly adaptive life-style of cyanobacteria that allows them to grow under diverse conditions. For example, the bloom-forming strain *Microcystis aeruginosa* PCC 7803 accumulates large amounts of glycolate when exposed to high-light stress (Meissner et al., 2015). The evolution of redundancy of an essential enzyme activity, such as PGPase, protects these organisms from damage by mutations as they frequently occur in non-stable environments. Alternatively, it is possible that at least some of these enzymes (such as Slr0586) have higher activity with substrates other than 2PG in *Synechocystis* and act as PGPase only when the main enzymes such as Slr0458 and Sll1349 are artificially inactivated. Redundant enzymes were also found for the downstream conversion of glycolate into glyoxylate (Eisenhut et al., 2008b) supporting the notion that the non-compartmented cyanobacteria need to avoid 2PG and glycolate accumulation to a larger extent than plants with their highly compartmented cellular metabolism.

## Author Contributions

MH, AK, and HB designed the study. SR performed the most experiments. JK performed the metabolome analysis. SL performed the enzyme assays. RK performed the phylogenetic analysis. SR, JK, AK, HB, and MH analyzed the data. SR and MH wrote the manuscript with contributions from all authors.

## Conflict of Interest Statement

The authors declare that the research was conducted in the absence of any commercial or financial relationships that could be construed as a potential conflict of interest.

## Footnotes

1 http://tree.bio.ed.ac.uk/software/figtree/

2 http://genome.annotation.jp/cyanobase/Synechocystis/genes/search?q=sll1349&keyword=search&m=gene_symbol%2Cgi_gname%2Cdefinition%2Cgi_pname

3 http://genome.microbedb.jp/cyanobase/

4 http://genome.microbedb.jp/CyanoBase

5 http://genome.annotation.jp/cyanobase/Synechocystis/genes/search?q=sll1349&keyword=search&m=gene_symbol%2Cgi_gname%2Cdefinition%2Cgi_pname

## Abbreviations

2PG: 2-phosphoglycolate
HAD: 2-haloacid dehalogenase
HC: high CO_2_
HCR: high-CO_2_-requiring
LC: low CO_2_ of ambient air
PCC: Pasteur culture collection
PGPase: 2PG phosphatase
WT: wild type
