# Supplementary material for: The Synechocystis sp. PCC 6803 Genome Encodes Up to Four 2-Phosphoglycolate Phosphatases
Source: Front Plant Sci. 2018 Nov 27;9:1718. doi: 10.3389/fpls.2018.01718 (PMC6278635; doi:10.3389/fpls.2018.01718)
Supplement: Supplementary file 1 [file Data_Sheet_1.pdf]

Supplementary data

Supplementary Table S1: Primers and antibiotics used in this work.

Supplementary Table S2: Similarities of cyanobacterial and Arabidopsis PGPase proteins

Supplementary Figure S1: Domain structure of putative PGPases

Supplementary Figure S2: Genotypes of the *Synechocystis* triple mutants

Supplementary Figure S3: Growth of *Synechocystis* mutants at ambient air on agar plates

Supplementary Figure S4: Expression and purification of *Synechocystis* PGPases in *E. coli*

Supplementary Figure S5: Alignment of *Synechocystis* PGPase-like proteins

Supplementary Table S1: Primers and antibiotics used in this work. Restriction site that were added to the gene-specific primers are underlined.

**Primers used for generation of *Synechocystis* mutants**			
**Gene**	**Primer**	**Antibiotic**	**Dosage**
slr0458	Fw_CCCAACTTATCCCAAAGATA<br>Rev_GCCGGAATTAGATTTACAAG	Chloramphenicol (Cm)	15 µg ml^{-1}
slr0586	Fw_CCCCTACAGCATTGACCGCAACATT<br>Rev_GGCAAACATTGGGTTCATCGGCTT	Erythromycin (Ery)	100 µg ml^{-1}
sll1349	Fw_AGATTTACAGAGCCHTTATT<br>Rev_GATTGAGCCAATATTTGAGG	Kanamycin (Kan)	50 µg ml^{-1}
slr1762	Fw_AGGGCAAGTTAGCGGAGTT<br>Rev_AAGGGCTATCTTCCCACGTT	Spectinomycin (Sp)	20 µg ml^{-1}
**Primers used for cloning in *E. coli* pET28a vector**			
slr0458	Fw_AACATATGGCTAACGAAAAAATTATTG<br>Rev_TTGAATTCTAAAGGGCGATCGCC		
slr0586	Fw_AACATATGGCTAATATGGTGCAGAAG<br>Rev_TTGAATTCTATTCAACCCCATGG		
sll1349	Fw_AACATATGGCCATTAAAGCTGTATTG<br>Rev_TTGAATTCTAGGATTTTAATGG		
slr1762	Fw_AACATATGGCTAATATCACCGTTCAAG<br>Rev_TTGAATTCTTAGGGCTGGCAGTG		

Supplementary Table S2: Identity and similarity matrix of *Synechocystis* PGPase candidate proteins. Similarities were given in brackets and calculated based on the Blosum62 substitution matrix.

	Slr0458	Sll1349	Slr1762	Slr0586	AtPGLP1
Slr0458	100%				
Sll1349	20(65)%	100%			
Slr1762	19(54)%	17(54)%	100%		
Slr0586	20(56)%	23(58)%	(17(48)%	100%	
AtPGLP1	14(29)%	10(24)%	11(23)%	20(32)%	100%

Supplementary Figure S1: Domain structure of putative PGPases

gi|499176709|Synechocystis sp. PCC 6803 slr0458

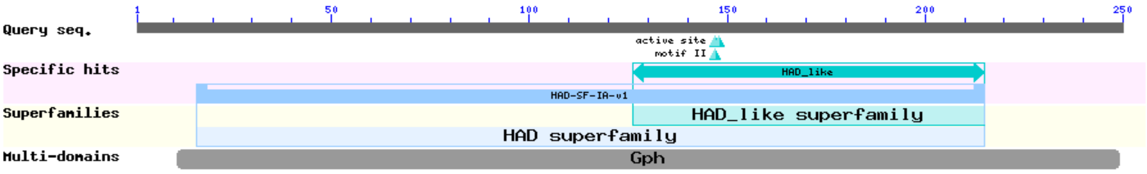

gi|499176735|Synechocystis sp. PCC 6803 slr0586

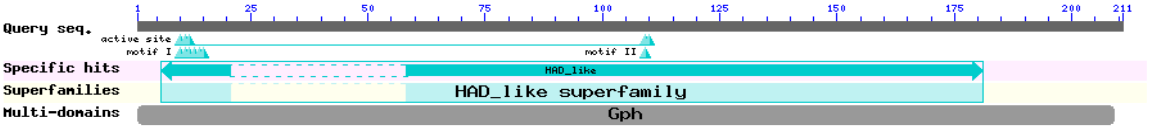

gi|499174608|Synechocystis sp. PCC 6803 sll1349

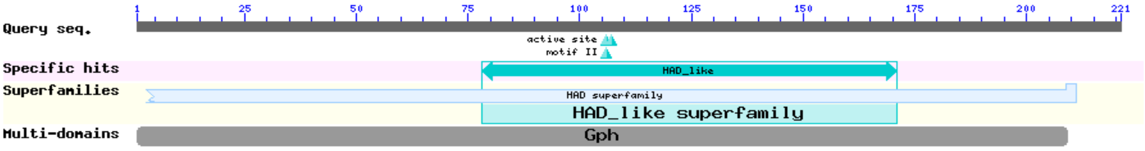

gi|499174101|Synechocystis sp. PCC6803 slr1762

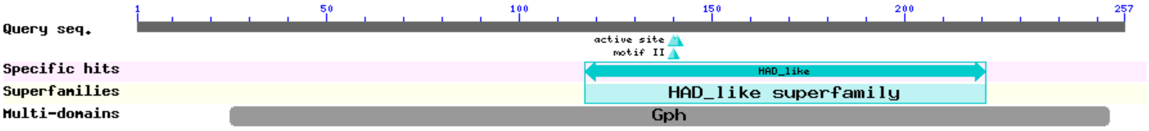

Figure S1: Domain structure including always a HAD-like family domain of the putative PGPases encoded by the ORF's of *slr0458*, *slr0586*, *sll1349,* and *slr1762* (domains were identified via the BLAST server at NCBI).

Supplementary Figure S2: Genotypes of the *Synechocystis* triple mutants

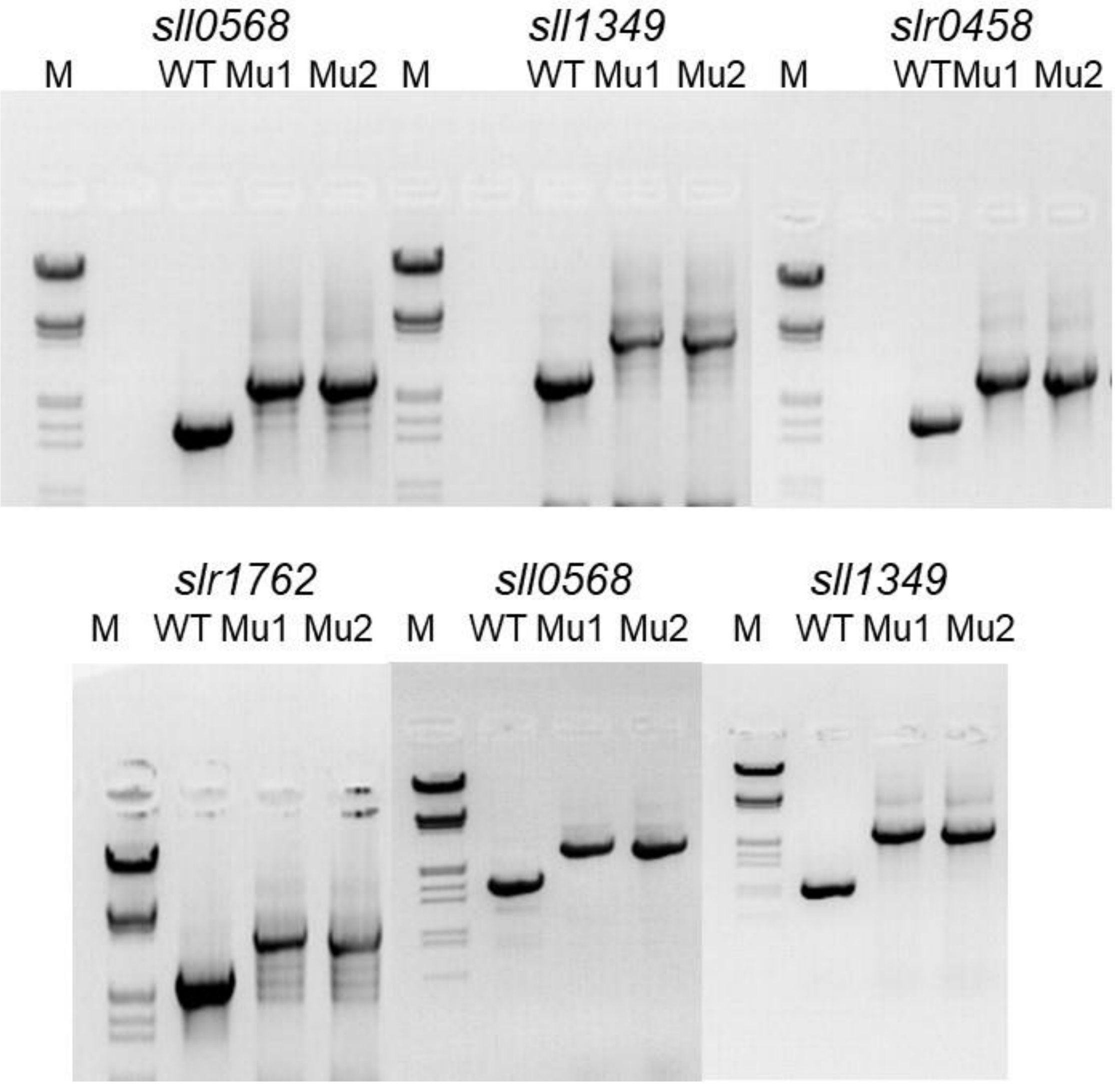

Figure S2: PCR reactions to proof the genotypes of two different triple mutants (upper panel: Δ*sll0568/sll1349/slr0458*, lower panel Δ*slr1762/sll0568/sll1349*) of *Synechocystis* defective in three of the four genes encoding for putative PGPases. Gene-specific primers were used (indicated above the pictures) and chromosomal DNA from two mutant clones (Mu1 and Mu2) as well as from wild type (WT) was used as template. M: DNA fragment size marker, λ-DNA cut with *Eco*RI and *Hin*dIII.

Supplementary Figure S3: Growth of *Synechocystis* mutants at ambient air on agar plates

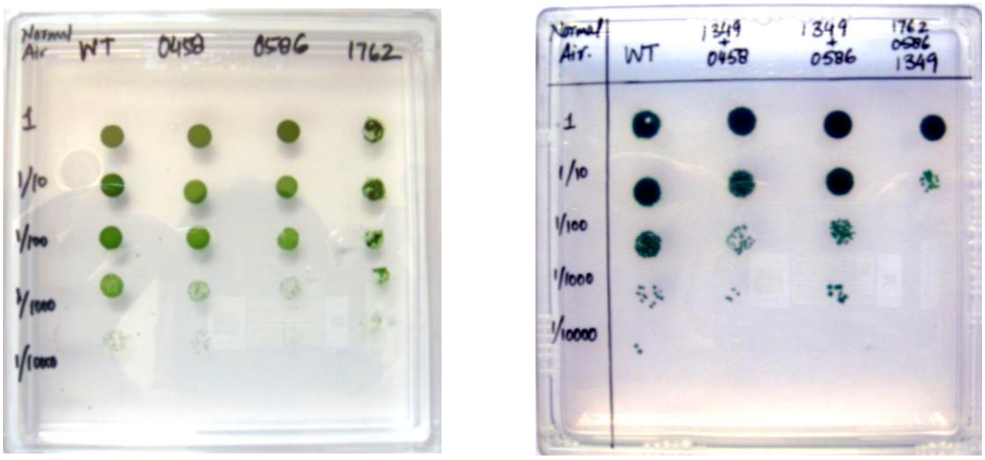

Figure S3: Left panel shows the growth of the *Synechocystis* wild type (WT) and single mutants Δ*slr0458*, Δ*slr0586*, and Δ*slr1762.* Right panel shows the growth of the *Synechocystis* wild type (WT), double mutants Δ*sll1349*/Δ*slr0458*, Δ*sll1349*/Δ*slr0586*, and triple mutant Δ*slr1762*/*slr0586*/*sll1349* (indicated by gene names). Increasingly diluted cell suspensions were dropped (2 µl each) on BG11 agar plates and incubated at 50 µmol photons m^{-2} s^{-1} at 30°C under ambient air conditions for 10 days.

Supplementary Figure S4: Expression and purification of PGPases in *E. coli*

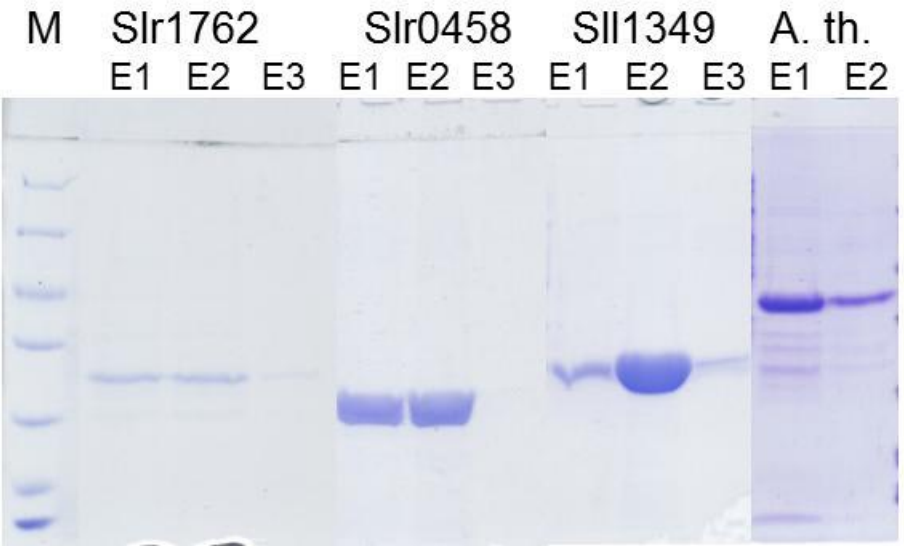

Figure S4: Purity of the recombinant PGPases from *Synechocystis* (Slr1762, Slr0458, Sll1349) and the PGPase from *Arabidopsis thaliana* (A.th.), which were used for the enzyme assays. The SDS-PAGE lanes show elution fractions (E) 1, 2, or 3 that were obtained after purification of the His-tagged proteins from recombinant *E. coli* cells. M: protein size marker (Biorad).

Supplementary Figure S5: Alignment of *Synechocystis* PGPase-like proteins, which was used for phylogenetic reconstruction.

```
                                   10        20        30        40        50        60        70
                           ....|....|....|....|....|....|....|....|....|....|....|....|....|....|
Synechocystis_PCC_6714     IVFDLDGVLIDSEEANYQAFAYGI--EQLGLPKPDR------------------QT-LISLVGLKAS--L
Synechocystis_PCC_6803     IVFDLDGVLIDSEEANYQAFAYGI--EQLGLPRPDR------------------QT-VVSLVGLKAS--L
Synechocystis_PCC_6803     LITDCDGPIMDVSDRYYQVYQYCL--EQVKLPGQDLNVLSKAEFWQRKRSKVPKAQ-IGLDSGLTKE--Q
Microcoleus_FGP-2          LITDFDGPIMDVSDRYYRVYQYCL--AETKRSDQAVRELPKAEFWDMKRARVPETE-IAILSGLDAD--Q
Microcystis_PCC_9701       LISDFDGPIMDVSERYYQVYQDCL--QKTAYKGQIIGELSKAEFWQLKRDQVSEKR-IGEMSGLDED--Q
Nostoc_PCC_7120            LITDFDGPIMDVSERYYRVYLFCL--EKTQHPGQPVRQLSKAEFWQMKRQHLPEKE-IARISGLDEA--Q
Synechocystis_PCC_6803     VLFDFDGTIADTHDAFFAIVNRLA--DEFGYPAVDK------------------TE-LARLKKLSSA--E
Microcoleus_FGP-2          IIFDFDGTLADTIDILLSITNRLS--AELGFKSATK------------------EQ-IAQLSNLTSW--Q
Microcystis_PCC_9701       VVFDFDGTIADTHDTFVEIVNRLA--KNFGYQPVNE------------------ED-LARLKNLSSQ--E
Nostoc_PCC_7120            IIFDFDGTIADTVDALVSIANRLA--VEFGYVQITP------------------EQ-LTLLRNFSSR--E
Synechocystis_PCC_6803     LIFDKDGTLENSKVYLEKLTVARLALLEQGIPAANF------------------GDRLAGAFGFDRSSAQ
Microcoleus_FGP-2          VIFDKDGTLEDSQEFLRLLGIKRSRLADAQIPG--V------------------GEPLQMAFGIEGG--S
Microcystis_PCC_9701       IVFDKDGTLEDSRAFWYDRAIARIQAIESRIPG--L------------------ESLLTKTFGIGAN--S
Nostoc_PCC_7120            ILFDKNGTLENSEAYLRSLGQKAARIVDAQVPG--I------------------GEPLLMAFGINGD--T
Arabidopsis                FIFDCDGVIWKG----------------------------------------------------------
Physcomitrella             FIFDCDGVIWKG----------------------------------------------------------
Zea                        FIFDCDGVIWKG----------------------------------------------------------
Escherichia_O157:H7        VAFDLDGTLVDSAPGLAAAVDMAL--YALELPVAGE------------------ER-VITWIGNGAD--V
Ralstonia                  VLIDLDGTLVDSAPDIVEAANRML--ADFGSPALPF------------------DT-VAGFIGRG-----
Rhodobacter_ATCC_17025     VVFDLDGTLVHSAPDIHAAVNRAL--AEEGAEPFSL------------------AE-ITGFIGNG-----

                                   80        90       100       110       120       130       140
                           ....|....|....|....|....|....|....|....|....|....|....|....|....|....|
Synechocystis_PCC_6714     MLEKLG----------------------CPAE------EGARIFRDFV-------------------QPF
Synechocystis_PCC_6803     MLEKLG----------------------CPRQ------EVERIFKDFV-------------------QPF
Synechocystis_PCC_6803     TEEFVA------------------------------------IRNR-T----------------------
Microcoleus_FGP-2          ARDFAQ------------------------------------KRRQ-T----------------------
Microcystis_PCC_9701       ARKFAH------------------------------------LRRQ-T----------------------
Nostoc_PCC_7120            AQEFSQ------------------------------------LRRQ-T----------------------
Synechocystis_PCC_6803     IIKYSQ----------------------ISPF------KIPFILKRFK----------------------
Microcoleus_FGP-2          LLQYSG----------------------ISIF------KFPLLIRRLK----------------------
Microcystis_PCC_9701       IIKQSQ----------------------VSPV------KIPFLLYRVK----------------------
Nostoc_PCC_7120            IIKYSG----------------------VSLI------KIPFLVKKVK----------------------
Synechocystis_PCC_6803     LDPGGLMAVGSRRDNVIAAA-------SYIAEQGYGWFQSLDIANQCF-------------------DQA
Microcoleus_FGP-2          IDPTGLLAVGSRRENEIVAA-------GYIAETGRGWLESLSIARRAF-------------------DEA
Microcystis_PCC_9701       LNPAGLMAVGSRRDNHIAAA-------GCIASTGRDWLTAMAIAKVAF-------------------QAA
Nostoc_PCC_7120            LDPAGLISVASRRETEIATA-------AYIAETGKGWFESLKIARQAL-------------------DDA
Arabidopsis                ----------------------------------------------------------------------
Physcomitrella             ----------------------------------------------------------------------
Zea                        ----------------------------------------------------------------------
Escherichia_O157:H7        LMERALTWARQERATLRKTMGKPPVDDDIPAE------EQVRILRKLF-------------------DRY
Ralstonia                  ---------------------------------------VPNLVRRVLETAGLTPRV-EAAEAVAMFHRH
Rhodobacter_ATCC_17025     ---------------------------------------VPVLIKRVLAARGETPDGHRHAEMQDRFMKH

                                  150       160       170       180       190       200       210
                           ....|....|....|....|....|....|....|....|....|....|....|....|....|....|
Synechocystis_PCC_6714     YIEN--LP--RLAAPMPQAATVLQSLKQRGYRILACTSGDRRTQTAALKGVG-L----------------
Synechocystis_PCC_6803     YIEN--LP--QLAAPMPNAVTVLTTLKQRDYRILACTSGDRRTQTAALQGVG-L----------------
Synechocystis_PCC_6803     -VHS--QPYLPYDQPLAGVEEILQDIKDRGIDLVLMTMRRESELQEALDRNH-F----------------
Microcoleus_FGP-2          -VHT--LPYMIYDQPVSGAVETLEKVQRAGVDLAVLTMRRVRELDEAFNRCN-L----------------
Microcystis_PCC_9701       -VHT--LPYLVHDRPVVGSLETLQKIQELKIDLVVMTMRRVSELDHAFNRHD-I----------------
Nostoc_PCC_7120            -VHT--EPYFQYDSPIPGALDVLLKVQQSGVDLVVMTMRRVWELDYAFQKYD-L----------------
Synechocystis_PCC_6803     -KEL--GKEIKDLKPYGDIKEVLTLLNQKGYVLGIVTSNSKDNVLSFLHNNG-L----------------
Microcoleus_FGP-2          -AEL--RSEIPNIQLFAGIKEVLLELKKLGFQLGIITSNSRENVLASLETNG-V----------------
Microcystis_PCC_9701       -REL--NKQIECLKPFHGWHQCLATLKERGYRLGIITSNTKENVTIFLDNNQ-L----------------
Nostoc_PCC_7120            -SEL--KNKIHELKPIPGIKEALLELKEHDYKLGIITSNSRENVTNFLSINE-L----------------
Synechocystis_PCC_6803     DRQIIANA--HTCPMFPGVVDSLTLWQQQGVKIAILSAARQRSVERFIADHQ-L----------------
Microcoleus_FGP-2          DEMF-KNS--AVSPLFVGSLEVLKYLSEAGLKLGILSAATTEVVQKFVKHHA-L----------------
Microcystis_PCC_9701       DEKV--PP--RLNPLYPQCLEVIRYLHAAGLQLAILSSDTTARVEQFVKENS-L----------------
Nostoc_PCC_7120            EKYI--GV--TPAPLFTGALEILQSLSNAGLKLGIVSAATTLEVKNFVAQHD-L----------------
Arabidopsis                ------------DKLIEGVPETLDMLRAKGKRLVFVTNNSTKSRKQYGKKFETLGLNVNEEEIFASSFAA
Physcomitrella             ------------DSLIEGVPETLDMLRSMGKRLVFVTNNSTKSRKQYGKKFESLGLSVSEEEIFASSFAA
Zea                        ------------DKLIDGVPETLDLLRSKGKRLVFVTNNSTKSRKQYGRKFETLGLSVDEEEIFASSFAA
Escherichia_O157:H7        YGEV--AE--EGTFLFPHVADTLGALQAKGLPLGLVTNKPTPFVAPLLEALD-I----------------
Ralstonia                  YAET--NG--RLGSVFPGVEAGLEALRRQGYRLACVTNKPRALAVPLLALTG-L----------------
Rhodobacter_ATCC_17025     YEAD--PT--ALTSVYPGAEAALRHLHSEGWRIALCTNKPYAASRQILSNFG-I----------------
```

```
                                220       230       240       250       260       270       280
                           ....|....|....|....|....|....|....|....|....|....|....|....|....|....|
Synechocystis_PCC_6714     -----WSAIEG-----------------------------------------------------------
Synechocystis_PCC_6803     -----WSAIET-----------------------------------------------------------
Synechocystis_PCC_6803     -----NHFFPID-RRYCLANDY------------------------------------------------
Microcoleus_FGP-2          -----GQFFPEN-RRYCLPNDY------------------------------------------------
Microcystis_PCC_9701       -----GRFFAAN-RRYCLNNNY------------------------------------------------
Nostoc_PCC_7120            -----GQFFPEN-RCYCLSNEY------------------------------------------------
Synechocystis_PCC_6803     -----EDIFAF-----------------------------------------------------------
Microcoleus_FGP-2          -----QDTFTF-----------------------------------------------------------
Microcystis_PCC_9701       -----LNLFDF-----------------------------------------------------------
Nostoc_PCC_7120            -----DSLFDF-----------------------------------------------------------
Synechocystis_PCC_6803     -----QSLVDV-----------------------------------------------------------
Microcoleus_FGP-2          -----GDYIQL-----------------------------------------------------------
Microcystis_PCC_9701       -----LNYIKI-----------------------------------------------------------
Nostoc_PCC_7120            -----GNYIQA-----------------------------------------------------------
Arabidopsis                AAYLQSINFPKDKKVYVIGEEGILKELELAGFQYLGGPDDGKRQIELKPGFLMEHDHDVGAVVVGFDRYF
Physcomitrella             AAYLKSIKFPSDKKVYIIGEAGIQLELKQAGINYIGGPEDGDKRIDLAPGQLMEHDHDVGAVVVGFDRYL
Zea                        AAYLQSIDFPKDKKVYVIGEEGILKELELAGFQYLGGPTDGDKKIELKPGLYMEHDEDVGAVVVGFDRYF
Escherichia_O157:H7        -----AKYFSV-----------------------------------------------------------
Ralstonia                  -----SQYLEV-----------------------------------------------------------
Rhodobacter_ATCC_17025     -----LDLFDA-----------------------------------------------------------

                                290       300       310       320       330       340       350
                           ....|....|....|....|....|....|....|....|....|....|....|....|....|....|
Synechocystis_PCC_6714     ---------------------------------------------MHAADD----SQFAKPDPRYLQELL
Synechocystis_PCC_6803     ---------------------------------------------MLAADD----SPFAKPDPRYLQQLL
Synechocystis_PCC_6803     ---------------------------------------------VKTGDT--------NDKPRLMERAL
Microcoleus_FGP-2          ---------------------------------------------VKTGDV--------KDKPLLMARAL
Microcystis_PCC_9701       ---------------------------------------------TKTNDV--------RDKTLLMAKAA
Nostoc_PCC_7120            ---------------------------------------------VKTRDI--------DDKPLLMQRAL
Synechocystis_PCC_6803     ---------------------------------------------VKAGTT----LF-GKNR--IINRVL
Microcoleus_FGP-2          ---------------------------------------------IYSGST-----F-GKHK--VINSWL
Microcystis_PCC_9701       ---------------------------------------------ICSGTP----LF-SKHK--IIDRLI
Nostoc_PCC_7120            ---------------------------------------------IYSGVT----IF-GKTT--IINNVL
Synechocystis_PCC_6803     ---------------------------------------------AKGSDQ-----GLSKPDPALYLLTC
Microcoleus_FGP-2          ---------------------------------------------EMGVDS-----TVHKPDPKLFLQAC
Microcystis_PCC_9701       ---------------------------------------------AQGCDR-----GLSKPDPLLLQETC
Nostoc_PCC_7120            ---------------------------------------------QVGVDD-----GPTKPDPILFLQAC
Arabidopsis                NYYKIQYGTLCIRENPGCLFIATNRDAVTHLTDAQEWAGGGSMVGALVGSTQREPLVVGKPSTFMMDYLA
Physcomitrella             NYYKLQYATLCIRENPGCMFIATNCDAVTHLTDAQEWAGGGSMVGAIKGSTKKEPLVVGKPSTFMMDYLA
Zea                        NYYKVQYGTLCIRENPGCLFIATNRDAVTHLTDAQEWAGGGAMVGALLGSTKQEPLVVGKPSTFMMDYLA
Escherichia_O157:H7        ---------------------------------------------VIGGDD----VQNKKPHPDPLLLVA
Ralstonia                  ---------------------------------------------LVAGDS----IAQMKPDPEPLRHAC
Rhodobacter_ATCC_17025     ---------------------------------------------IVGGDC----LPQRKPDPAPLRAAA

                                360       370       380       390       400       410       420
                           ....|....|....|....|....|....|....|....|....|....|....|....|....|....|
Synechocystis_PCC_6714     AP--YDYQTLLHVEDA-EVGIRMGQACGAV-SIFAEYGYGSLPA--D----LPVDYRLTQLADIL-----
Synechocystis_PCC_6803     AP--YDYQTLLHVEDA-EVGIRMGQACGAV-SIFAEYGYGSLPA--D----LPVDHRLSQLADIL-----
Synechocystis_PCC_6803     AELP-PADSVWMVGDT-EADILAAQRGNLP-AIAVLSGIRNREQLER----YQPDFIVDNLAEAVNLFYQ
Microcoleus_FGP-2          AELP-PASDIWMVGDT-EADIVSAKTHGVK-VIGVLCGIRDRTQLEK----HQPDLIANNLSEAVEIIL-
Microcystis_PCC_9701       KELP-AAADTWMVGDT-EADIAAAKSQNIK-VIGVLSGIRSRSRLES----YEPDYIVNNLGEAVDVILG
Nostoc_PCC_7120            AELP-PAADTWMVGDT-EADITAAKKHGVK-VIAVESGIRDRTRLQQ----YHPDLIVQNLSAAVDIILE
Synechocystis_PCC_6803     KEHKFGTDEVIYVGDE-TRDISAAKKSRLT-MVSVAWGFSPPAILQE----YEPDFLVHQPKELLTAIAS
Microcoleus_FGP-2          KTEHINPKQVVYVGDE-IRDIEAARKTGIK-VIAVAWGFNSQEALAA----HNPDFLIKRPQELIEIMS-
Microcystis_PCC_9701       RQNKFCPDEMIYVGDE-TRDITAAQKSQVQ-VVAVAWGFNSPQILTQ----FNPDHLIHHPLELLDILD-
Nostoc_PCC_7120            RQKQFKPQSVIYVGDE-TRDIEASKKANIK-VIAVTWGFNSPEILAK----QNPDFLIHQPRELLEVIK-
Synechocystis_PCC_6803     RELGVKPEHTLMIGDA-QGDITMAKGAHAQGAIAIHWPGYAQGN--L----VGTDATIADLQQI------
Microcoleus_FGP-2          EKLGVTPAATLMVGDS-AGDIQMGKNAGAAGCIGICWGNAQVSY--L----ENADVAIASLDEIK-----
Microcystis_PCC_9701       QALGTAVDKTLMVGDT-RADWEMAKQAKSAAAIAISWQPETHQD--L----QLADVVIRELTAIS-----
Nostoc_PCC_7120            QTLGVEPEATLMVGDA-VGDMQMARNAQAAGCIGITWV-NKPNN--V----QGANVVINQLDEIQ-----
Arabidopsis                DKFGIQKSQICMVGDRLDTDILFGQNGGCK-TLLVLSGIT------------------------------
Physcomitrella             SEFNIKTSQICMVGDRLDTDILFGQNGGCA-TLLVLSGVTTLETLQSPENKIQPDFYTTKISDLLAAK--
Zea                        KKFGITTSQICMVGDRLDTDILFGQNGGCK-TLLVLSGVTSLQTLQSPDNSIQPDFYTNQISDFLTL---
Escherichia_O157:H7        ERMGIAPQQMLFVGDS-RNDIQAAKAAGCP-SVGLTYGYNYGEAIDL----SQPDVIYQSINDLLPALGL
Ralstonia                  NLLDVDTAQGVLVGDS-AVDVAAARAAGIP-VCLVRYGYAGPGGPAA----LGADALLDSLEALPALLTP
Rhodobacter_ATCC_17025     A--ALTEEVVLYVGDS-EVDAATAEAAGLR-FALFTEGYRHAPVHDL-----PHHGLFSHHDELPDLLR-
```

```
                                  430
                          ....|....|.
Synechocystis_PCC_6714    -------AIAT
Synechocystis_PCC_6803    -------AIAL
Synechocystis_PCC_6803    ------HHGVE
Microcoleus_FGP-2         -------GHKN
Microcystis_PCC_9701      -----SLRAIG
Nostoc_PCC_7120           -----SSVVKI
Synechocystis_PCC_6803    LDNHRWYPLKS
Microcoleus_FGP-2         ------NLWEV
Microcystis_PCC_9701      --------RAG
Nostoc_PCC_7120           --------NSQ
Synechocystis_PCC_6803    -------HCQP
Microcoleus_FGP-2         -------ILAD
Microcystis_PCC_9701      -------VISF
Nostoc_PCC_7120           -------ILES
Arabidopsis               ---------NL
Physcomitrella            ------KVASA
Zea                       ------KAATV
Escherichia_O157:H7       PHSENQESKND
Ralstonia                 ---A-RLAPAA
Rhodobacter_ATCC_17025    -------HLLA
```
